# Potential protective effects of Huanglian Jiedu Decoction against COVID-19-associated acute kidney injury: A network-based pharmacological and molecular docking study

**DOI:** 10.1515/med-2023-0746

**Published:** 2023-07-06

**Authors:** Weichu Wu, Yonghai Zhang, Guoyuan Liu, Zepai Chi, Aiping Zhang, Shuying Miao, Chengchuang Lin, Qingchun Xu, Yuanfeng Zhang

**Affiliations:** Department of Urology, Shantou Central Hospital, Shantou, 515031, PR China; School of Integrative Medicine, Gansu University of Traditional Chinese Medicine, Lanzhou, 730000, PR China; Department of Urology, The First Affiliated Hospital, Zhejiang University School of Medicine, Hangzhou, 310003, China; Department of Traditional Chinese Medicine, Shantou Central Hospital, Shantou, 515031, PR China

**Keywords:** COVID-19, Huanglian Jiedu Decoction, acute kidney injury, network pharmacology

## Abstract

Corona virus disease 2019 (COVID-19) is prone to induce multiple organ damage. The kidney is one of the target organs of SARS-CoV-2, which is susceptible to inducing acute kidney injury (AKI). Huanglian Jiedu Decoction (HLJDD) is one of the recommended prescriptions for COVID-19 with severe complications. We used network pharmacology and molecular docking to explore the therapeutic and protective effects of HLJDD on COVID-19-associated AKI. Potential targets related to “HLJDD,” “COVID-19,” and “Acute Kidney Injury/Acute Renal Failure” were identified from several databases. A protein–protein interaction (PPI) network was constructed and screened the core targets according to the degree value. The target genes were then enriched using gene ontology and Kyoto Encyclopedia of Genes and Genomes. The bioactive components were docked with the core targets. A total of 65 active compounds, 85 common targets for diseases and drugs were obtained; PPI network analysis showed that the core protein mainly involved JUN, RELA, and AKT1; functional analysis showed that these target genes were mainly involved in lipid and atherosclerosis signaling pathway and IL-17 signal pathway. The results of molecular docking showed that JUN, RELA, and AKT1 had good binding activity with the effective chemical components of HLJDD. In conclusion, HLJDD can be used as a potential therapeutic drug for COVID-19-associated AKI.

## 1 Introduction

Novel coronavirus pneumonia (corona virus disease 2019, COVID-19), caused by a novel coronavirus (SARS-CoV-2), has resulted in catastrophic human deaths and economic losses throughout the world, posing a significant danger to global public health [1,2]. The clinical manifestations of COVID-19 range from asymptomatic or mild infection to rapidly progressive acute respiratory distress syndrome (ARDS), which predisposes to multi-organ complications such as acute renal insufficiency (acute kidney injury [AKI]) [3]. Evidence shows that coronaviruses employ angiotensin-converting enzyme II (ACE2) as a cellular receptor to invade human cells and that ACE2 is highly expressed in the kidney, one of the coronavirus’s target organs and one of the most often damaged extra-pulmonary organs [4]. Approximately 36.6% of confirmed patients have AKI [5]. AKI is positively associated with increased in-hospital mortality in patients [6]. Currently, there is no specific clinical treatment for COVID-19-associated AKI, and the main treatment options include general therapy, supportive therapy, and renal replacement therapy [5,6]. The main Western drugs used for the treatment of COVID-19 are raltegravir, famipiravir, and chloroquine. The US Food and Drug Administration granted Pfizer’s Paxlovid (nirmatrelvir and ritonavir copackaged for oral use) an Emergency Use Authorization in December 2021 for the treatment of mild-to-moderate COVID-19 patients. Clinical studies have found that Paxlovid is effective in early interventions, reducing the risk of serious illness and death [2,7]. However, there is insufficient evidence on whether Paxlovid is effective in severe or advanced conditions. Although a number of vaccines have been successfully developed, their effectiveness decreases as the virus mutates. Traditional Chinese medicine (TCM) has been successfully treating infectious and non-infectious diseases for thousands of years in China. During the 2019 COVID-19 outbreak in Wuhan, the Chinese national government appointed TCM experts to screen and develop effective TCM prescriptions for COVID-19 [8]. To date, eight versions of new coronavirus diagnostic and treatment protocols have been published by the National Health Council and the State Administration of Traditional Chinese Medicine. Clinical use of TCM prescriptions and herbs has a significant impact on COVID-19 by relieving symptoms, delaying disease progression, improving cure rates, and reducing mortality [1,5,8]. Chinese medicine plays an important role in preventing morbidity and reducing mortality in patients with COVID-19 [9,10].

In the published pneumonia diagnosis and treatment protocol for novel coronavirus infection (Trial Operation Third Edition), Huanglian Jiedu Decoction (HLJDD) was officially formally included [10]. This prescription is composed of four herbs, namely Huang Lian, Huang Qin, Huang Bai, and Gardenia in a ratio of 3:2:2:3. Patients with the following clinical symptoms may benefit from HLJDD: high fever, cough, little or yellow sputum, chest tightness, shortness of breath, and bloating. HLJDD which was used for treating “heat-syndrome,” was first described in Medical Secrets of an Official “Wai Tai Mi Yao,” by WANG TAO, a medical scientist in the Tang Dynasty [10,11]. Studies have shown that HLJDD has obvious anti-bacterial, anti-fungal, anti-viral, anti-endotoxin, anti-inflammatory, and immunomodulatory effects, which can effectively alleviate the immunosuppressive effects of hormonal drugs and is an effective drug in the treatment of acute infectious diseases [11–14]. Recent studies have found that the active compounds in HLJDD may have therapeutic effects on COVID-19 by targeting vascular endothelial growth factor A, INS, and other genes that regulate multiple signaling pathways [15]. In addition, HLJDD can effectively inhibit LPS-induced AKI in mice [16]. Animal studies reveal that HLJDD may exert protective effects against diabetic nephropathy by ameliorating disorders of glucolipid metabolism and renal injury [17]. Previous studies have found quercetin to be one of the active components of HLJDD. Quercetin could be an effective drug to improve AKI by regulating M1/M2 macrophage polarization and Mincle/Syk/NF-κB signaling-mediated macrophage inflammation [18–20]. Based on these findings, HLJDD may have a protective effect against COVID-19-associated AKI.

Network pharmacology is based on the “disease–gene–target–drug” interaction network [21]. Through the network analysis system, we can observe the intervention and influence of drugs on the disease network, and reveal the mystery of the synergistic effect of multi-molecular drugs on the human body. It is similar to the principle of multi-component, multi-pathway, and multi-target synergy of Chinese medicine and its compound [22,23].

In this study, network pharmacology and molecular docking technologies were utilized to investigate the mechanism of HLJDD in the treatment of COVID-19-associated AKI and to provide a theoretical basis for its clinical application.

## 2 Materials and methods

### 2.1 Collection of drug chemical components and screening of potential targets for drug (HLJDD)

The active chemical components and drug targets of HLJDD were obtained through the TCM System Network Pharmacology Database [24] (http://ibts.hkbu.edu.hk/LSP/tcmsp.php). For the Database, TCMSP^TM^ was selected and the search entry was set to herb name. In the screening of the active chemical components of HLJDD, the drug composition of the compound was entered under the search entry: “Huanglian (Coptidis Rhizoma),” “Huangqin (Scutellariae Radix),” “Huangbo (Phellodendri Chinensis Cortex),” and “Zhizi (Gardeniae Fructus).” The screening conditions were set as oral bioavailability ≥30% and drug-likeness (DL) ≥0.18 [25]. DL is commonly used to assess the possible failure properties of a compound. The active chemical components and target information obtained from the database were standardized in the format with Drugbank [26] (https://www.drugbank.ca/).

### 2.2 Screening of potential targets for disease (AKI/acute renal failure (ARF) and COVID-19)

Based on the Genecards Human Genetic Database [27] (https://www.genecards. org/), Online Mendelian Inheritance in Man [28] (https://www.ncbi.nlm.nih.gov/omim), TTD database (http://db.idrblab.net/ttd/), “Acute Kidney Injury (AKI)/Acute Renal Failure (ARF)” and “COVID-19” were searched as the keywords. The disease data were merged and de-duplicated, and the intersection of the two disease targets after merging and de-duplication was performed with the help of the Venn package in R software 3.6.0 [29], and the intersection result was used as the final disease target.

### 2.3 Screening of potential targets for drug–disease

Venn diagramming with an online program was used to match the drug targets obtained in “1.1” and “1.2” for HLJDD with the targets of COVID-19-associated AKI. The target genes were obtained which were used as potential targets of the herbal compound for the treatment of the disease, and then the Venn diagrams were drawn.

### 2.4 Construction of chemical composition–target network diagram

The targeted genes obtained in “1.3” were imported into the network visualization software Cytoscape 3.8.0 to construct a chemical composition–target interaction network. The network was analyzed and the degree value parameters were used as a criterion to evaluate the importance of nodes in the network.

### 2.5 Construction of the protein–protein interaction (PPI) network

To obtain the relationship on protein interactions, the cluster of targeted genes obtained from “1.3” was imported to the String database (https://string-db.org/), “homo sapiens” was specified as the present setting, and the confidence level was set to 0.4. The output was saved as a TSV file and loaded into Cytoscape 3.8.0 to create a PPI network.

### 2.6 Analysis of gene ontology (GO) enrichment and Kyoto Encyclopedia of genes and genomes (KEGG) pathways

Enrichment analysis of GO and KEGG pathway analysis was performed. The GO enrichment analysis includes cell component (CC), molecular function (MF), and biological process (BP). The target proteins obtained after weight loss at the predicted target sites were imported into the GO database. A *p*-value ≤0.05 was considered significant. The KEGG route was examined using the Reactome Pathway Database (https://reactome.org/Pathway Browser/) after the GO function had been annotated. The required Biocmanager and other program packages were installed at the Bioconductor (https://www.bioconductor.org/) website. Bar plots and bubble charts were plotted.

### 2.7 Molecular docking

Preparation of molecule ligands file: according to the core protein obtained from “1.5” by “1.4” chemical composition–target network diagram to match the corresponding ligand, 3D crystal structure of drug molecule structures were obtained from PubChem databases (https://pubchem.ncbi.nlm.nih.gov/compound/) and exported to a ligand file in PDBQT format. Preparation of protein receptor file: based on the previous network pharmacology screening findings, the target protein’s 3D structure was obtained from the protein database (PDB) (http://www.rcsb.org/). Molecule ligands and protein receptors were saved in PDBQT format with AutoDock. To specify the molecular docking range, the PDBQT structures of the receptor and ligand were imported into AutoDock. Finally, molecular docking was performed using AutoDock and R software to select the binding mode with the lowest free energy. Binding energy less than 0 indicates that the ligand and receptor molecules can bind spontaneously. The more negative the energy the better the ligand.

**Figure 1:**
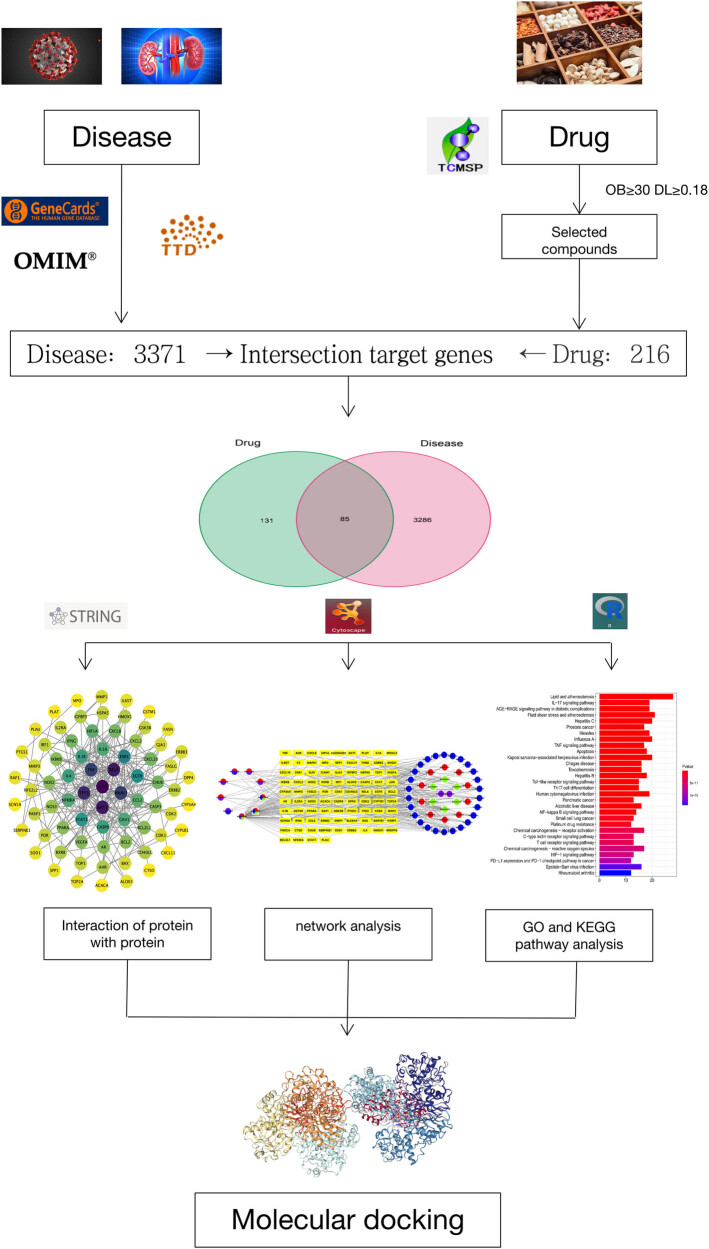
Analysis process of this study.


The schematic illustration of this study is shown in Figure 1.

**Ethical approval:** The conducted research is not related to either human or animals use.

## 3 Results

### 3.1 Compound chemical composition and drug targets

After merging and de-duplication, a total of 65 drug chemical components of HLJDD were collected and 216 predicted targets were identified in this study.

### 3.2 Disease targets

**Figure 2:**
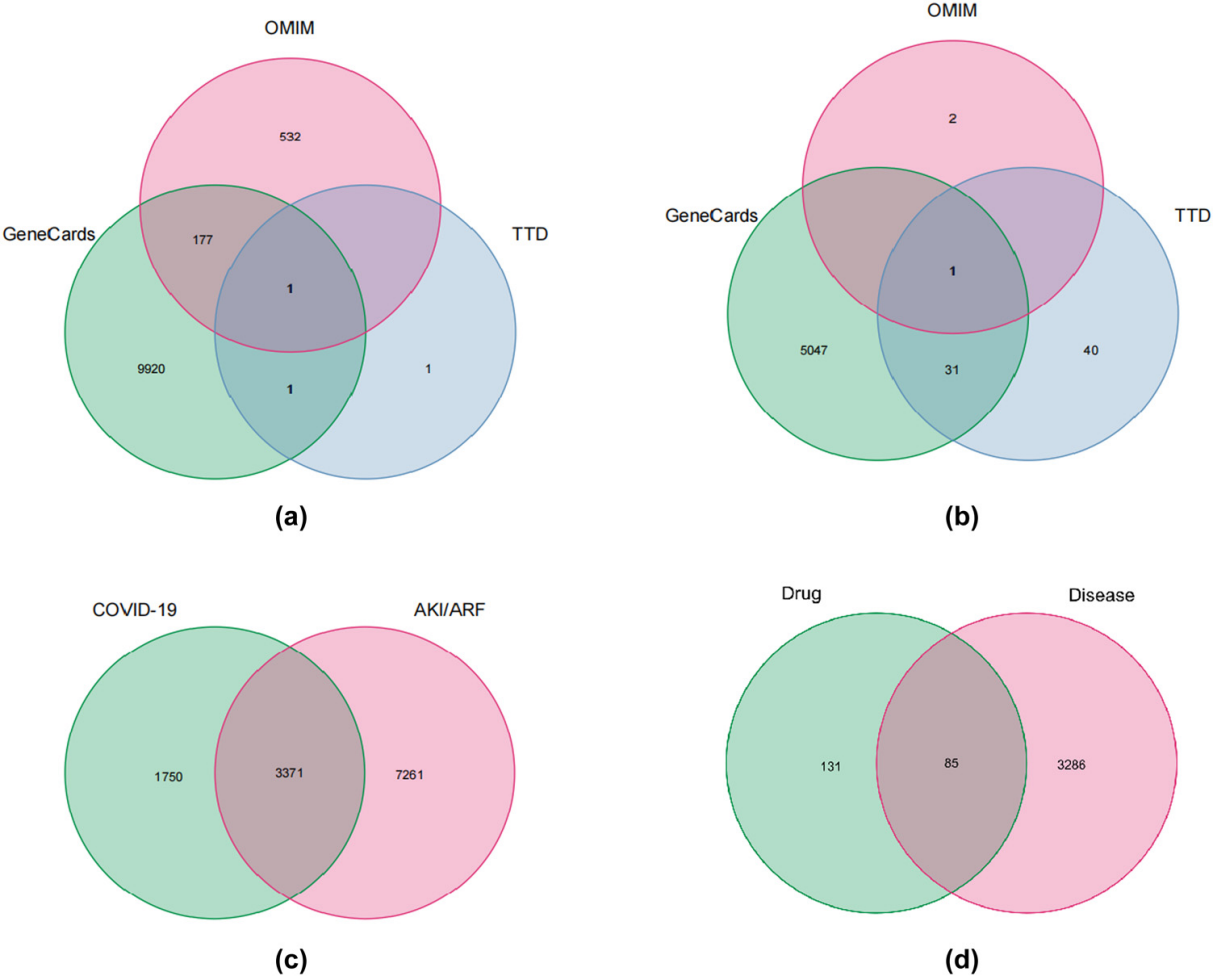
Potential HLJDD-related target genes in COVID-19-associated AKI: (a) AKI/ARF targets obtained from Genecards, OMIM, and TTD, (b) COVID-19 targets obtained from Genecards, OMIM, and TTD, (c) COVID-19-associated AKI/ARF targets, and (d) Venn diagram of the intersection relationship of target genes between HLJDD, AKI/ARF, and COVID-19.

The GeneCards, OMIM, and TTD databases were searched with “Acute Kidney Injury/Acute Renal Failure” and “COVID-19” as keywords, respectively. The results were summarized by the “Venn” program package of R software. The disease targets obtained from the three databases were summarized and duplicates were removed (Figure 2a and b). A total of 3,371 disease targets were obtained, which were corrected by the UniProt database and used as the final source of candidate disease targets, as shown in Figure 2c.

### 3.3 Drug–disease target genes

The Venn diagram program package was installed at the Bioconductor (https://www.bioconductor.org/) website. The compound drug targets and disease targets obtained in “1.1” and “1.2” were imported into the R software, and the target genes were obtained and Venn diagrams were drawn. A total of 85 drug–disease target genes were obtained (Figure 2d).

### 3.4 Construction of compound active compounds–target gene network map

**Figure 3:**
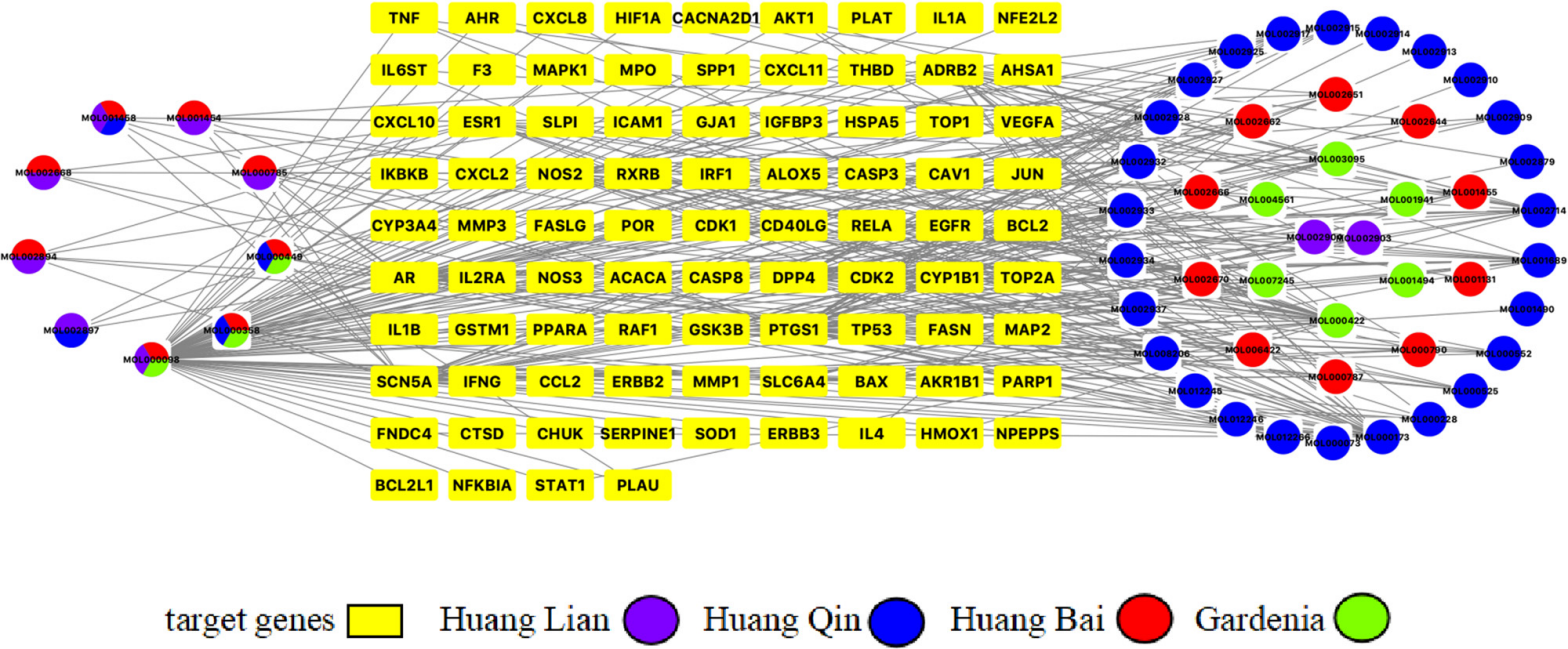
Compounds–target gene network. The target genes are shown as yellow rectangles and the active chemical components of the compound are shown as circles. Different drugs are shown as different colors, among which Huang Lian is shown as purple, Huang Qin is shown as blue, Huang Bai is shown as red, and Gardenia as green.

Compounding active chemical components–target action network was constructed by using the Import Network plugin in Cytoscape 3.8.0 (Figure 3). The relationship between compounds and targets in the network was shown with each edge. A chemical component often corresponds to multiple target genes, and a single target gene also corresponds to multiple chemical components at the same time. The chemical components were subordinated to different herbal medicines, which indicated that the process of HLJDD treating diseases was multi-component and multi-target, which also coincided with the complexity of the role of TCM.

### 3.5 PPI networks

**Figure 4:**
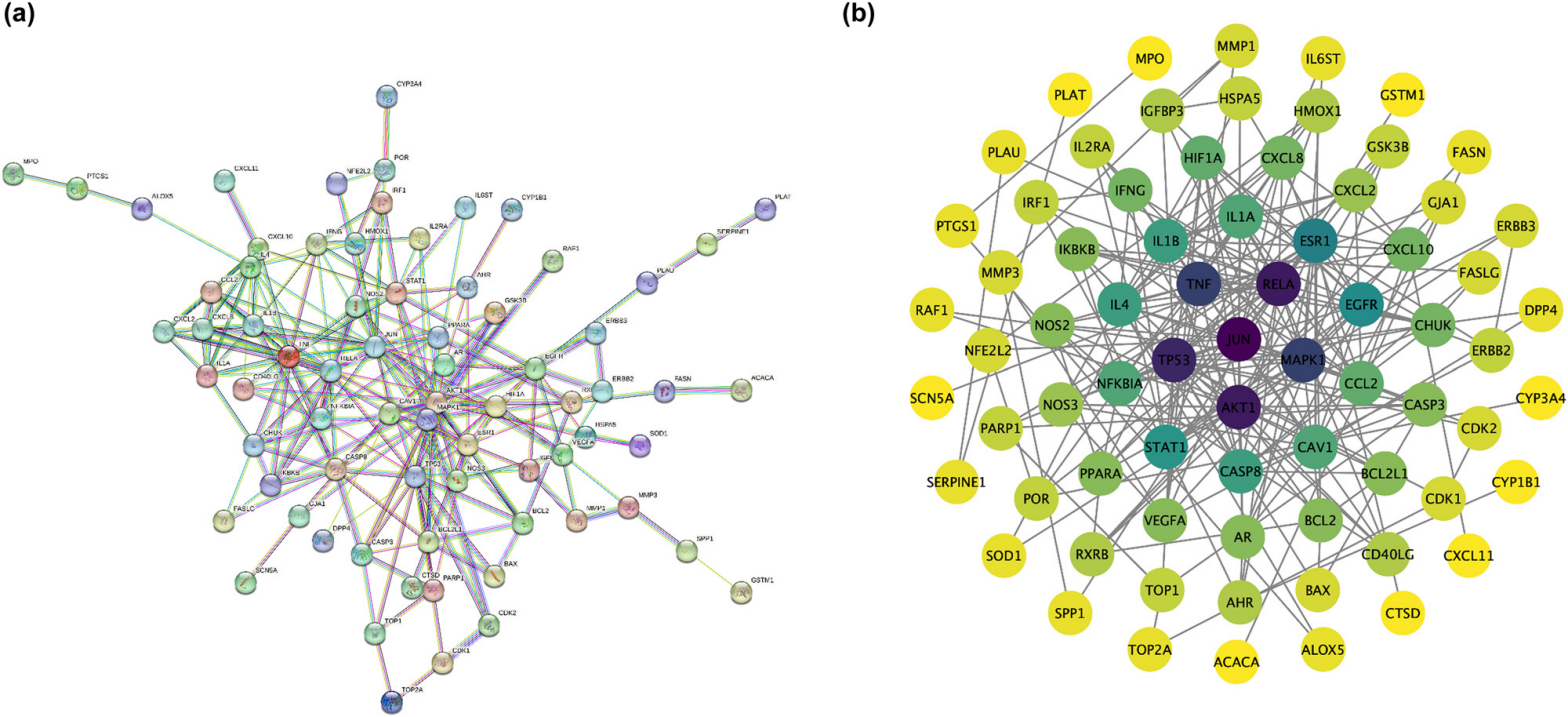
PPI network of potential target genes related to HLJDD on COVID-19-associated AKI. (a) PPI network: nodes represent proteins and edges represent protein–protein action relationships. (b) Core HLJDD-COVID-19-associated AKI PPI network. The core protein screening parameters included: Betweenness Centrality, Closeness Centrality, Degree Centrality, Eigenvector Centrality, and Local Average Connectivity-based method (LAC).

The string_interactions.txt file obtained from the STRING database was imported to Cytoscape 3.8.0, the network analyze plugin was applied to construct the network and the CytoNCA plugin was selected to perform network topological analysis and screen the key nodes in the network (Figure 4a). The concentric network diagram of core proteins was constructed by screening the core proteins according to the core protein screening parameters (Figure 4b).

The degree is one of cytoHubba’s topological analysis approaches. The higher the degree of network connection indicates the closer the relationship between proteins. Each node displays a distinct color depth based on its degree. The higher the degree, the darker the color. The average degree was 22.52. There were three target genes (JUN, RELA, and AKT1) with a greater degree of significance, and they were identified as potentially relevant genes involved in the processes of HLJDD therapy of COVID-19-associated AKI (Table 1).

**Table 1:** Protein topological analysis

Number	Protein	Betweenness	Closeness	Degree	Eigenvector	LAC
1	JUN	1160.072766	0.514285714	24	0.326208085	5.5
2	RELA	520.3579831	0.486486486	22	0.326378465	6.272727273
3	AKT1	747.7982437	0.486486486	22	0.222518116	2.636363636
4	TP53	873.4090544	0.496551724	21	0.241612837	4.380952381
5	MAPK1	675.1123282	0.489795918	19	0.228730246	3.789473684
6	TNF	259.542224	0.447204969	19	0.277028799	6.210526316
7	ESR1	375.0596077	0.461538462	14	0.187064067	4
8	EGFR	621.1730494	0.436363636	13	0.125641063	2.615384615
9	STAT1	325.0209183	0.45	12	0.155459821	2.166666667
10	IL4	437.6491267	0.393442623	11	0.172031865	5.090909091
11	CASP8	151.7557781	0.413793103	11	0.163645267	3.272727273
12	IL1B	45.10818883	0.397790055	11	0.18860589	6.545454545
13	CAV1	237.617026	0.43902439	10	0.104360625	1.4
14	IL1A	34.64010251	0.389189189	10	0.174545407	6.4
15	NFKBIA	47.3953072	0.43373494	10	0.181601599	4.6
16	HIF1A	103.6371202	0.455696203	9	0.162191033	4.888888889
17	CCL2	20.49008427	0.385026738	9	0.163622051	6.666666667
18	CHUK	48.01707653	0.380952381	8	0.133682579	3.5
19	CXCL10	154.3555389	0.36	8	0.109749712	3.75
20	CXCL8	13.88605382	0.382978723	8	0.15461652	6.25

### 3.6 GO enrichment analysis

**Figure 5:**
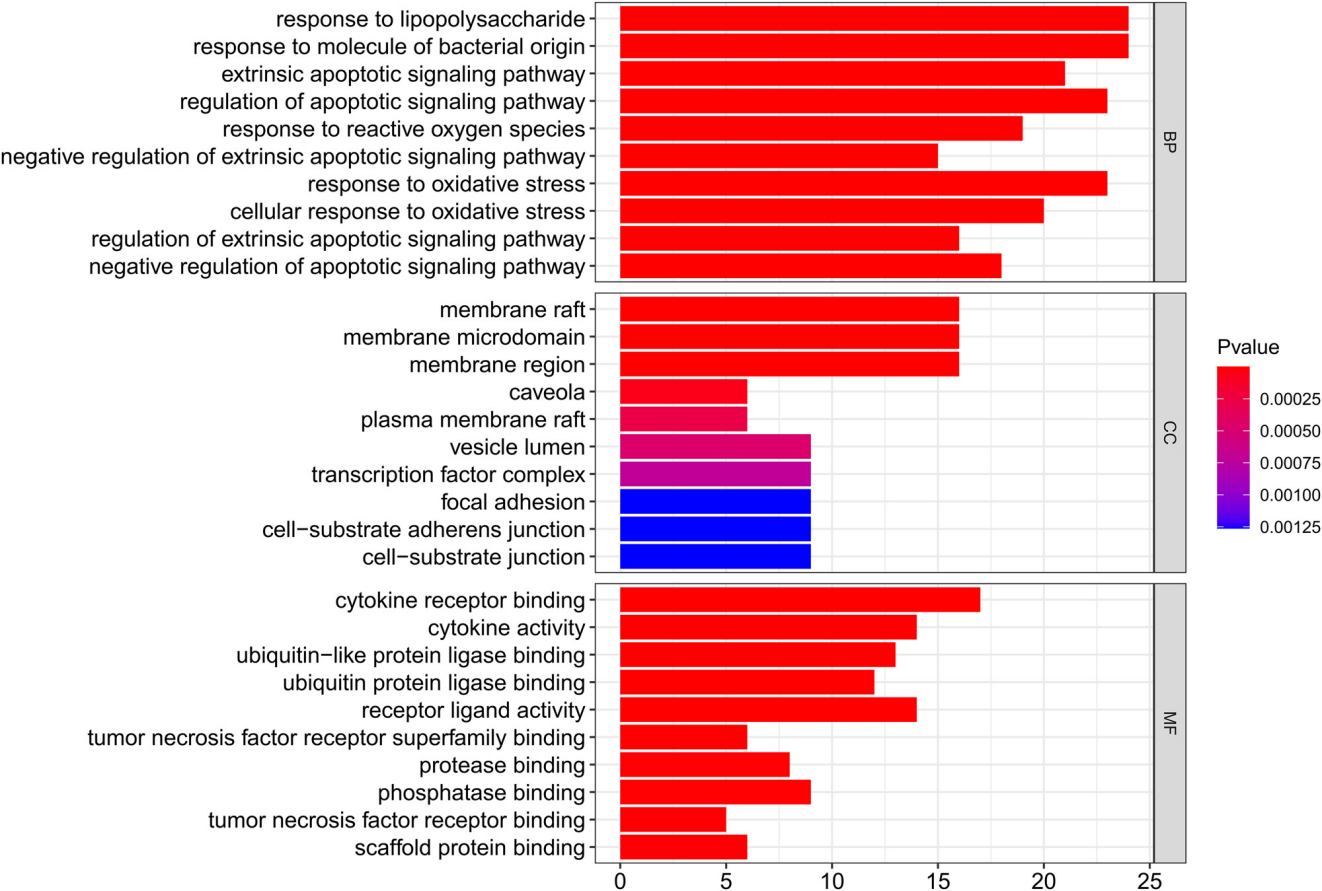
Top ten entries of the GO enrichment of screened target genes in COVID-19-associated AKI. The horizontal coordinates indicate the number of enriched genes, BP represents biological processes, CC represents cell components, and MF represents molecular functions. The color indicates the *p-*value.

The GO functional annotation of HLJDD and the pathway analysis of reactome were both annotated with the GO database. The top ten enriched entries of each rank were selected, and bar plots were drawn according to the *p*-value of each item and the number of genes enriched on them. The enrichment conditions were *p*-value <0.05 and *Q*-value <0.05, and the rest was set by default. The GO analysis had shown 1,714 entries on BP, 59 entries on CC, and 160 entries on MF with *p* < 0.05 (Figure 5).

### 3.7 KEGG pathway analysis

**Figure 6:**
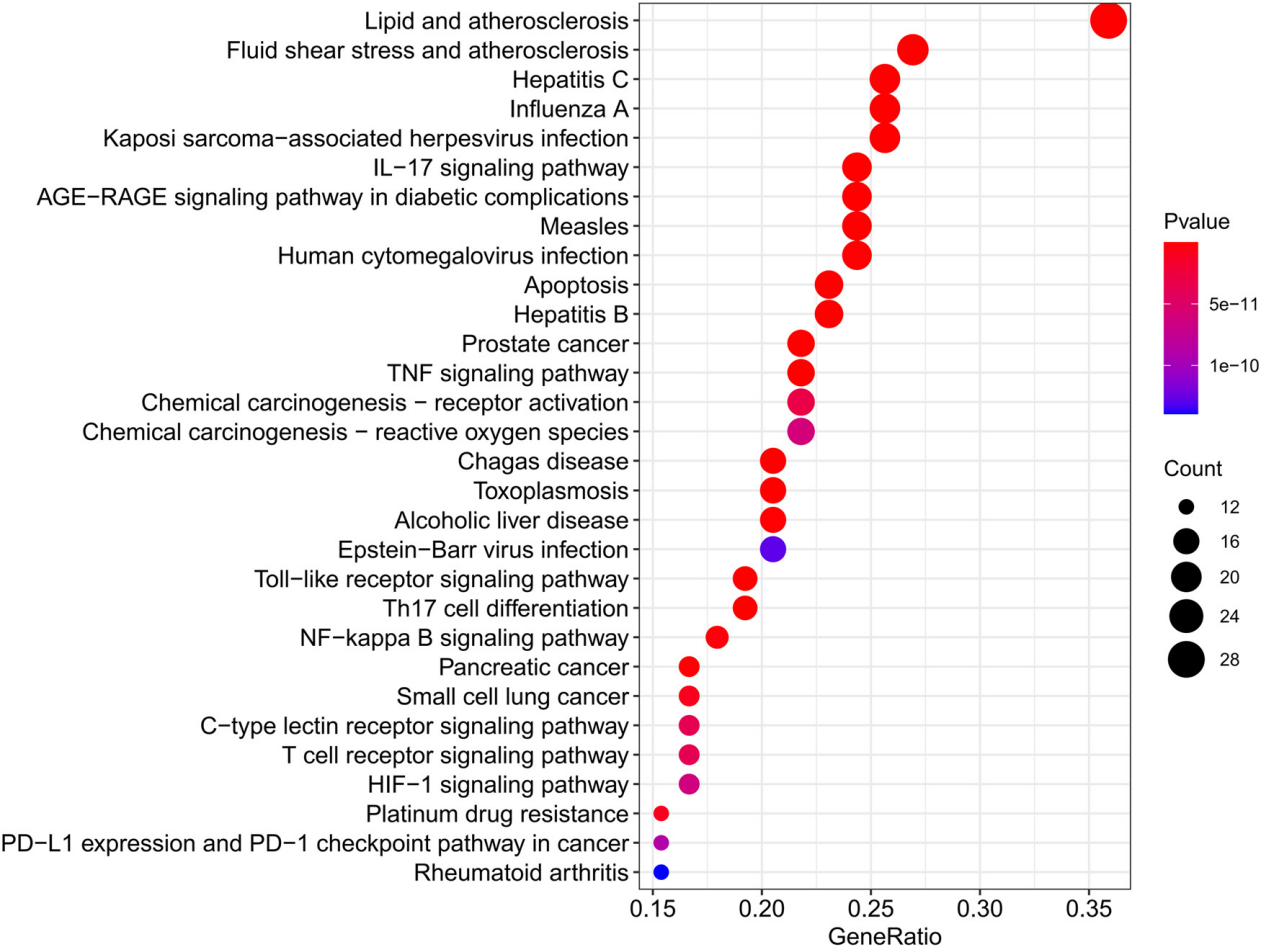
Top 20 potential KEGG pathway enrichment of target genes in COVID-19-associated AKI. The horizontal coordinates indicate the index of GeneRatio, while the vertical coordinates indicate KEGG enrichment entries. The index of GeneRatio represents the ratio of the number of pathway-related target genes, and it represents the number of annotated genes in certain pathways, the higher the score of GeneRatio, the higher the level of enrichment. The size of the dots represents the number of target genes in their representative pathways. The more the genes involved, the larger the bubble. The color of the dot presents the different *p-*values.

KEGG pathway analysis was performed. The results of the analysis showed that 85 compound disease target genes were enriched to a total of 152 pathways (*p* < 0.05). The top 20 pathways were selected, and the bubble plots were plotted according to the *p*-value of each pathway and the number of genes enriched on it (Figure 6). The significance of the *p*-value is the same as described earlier.

### 3.8 Molecular docking validation

**Figure 7:**
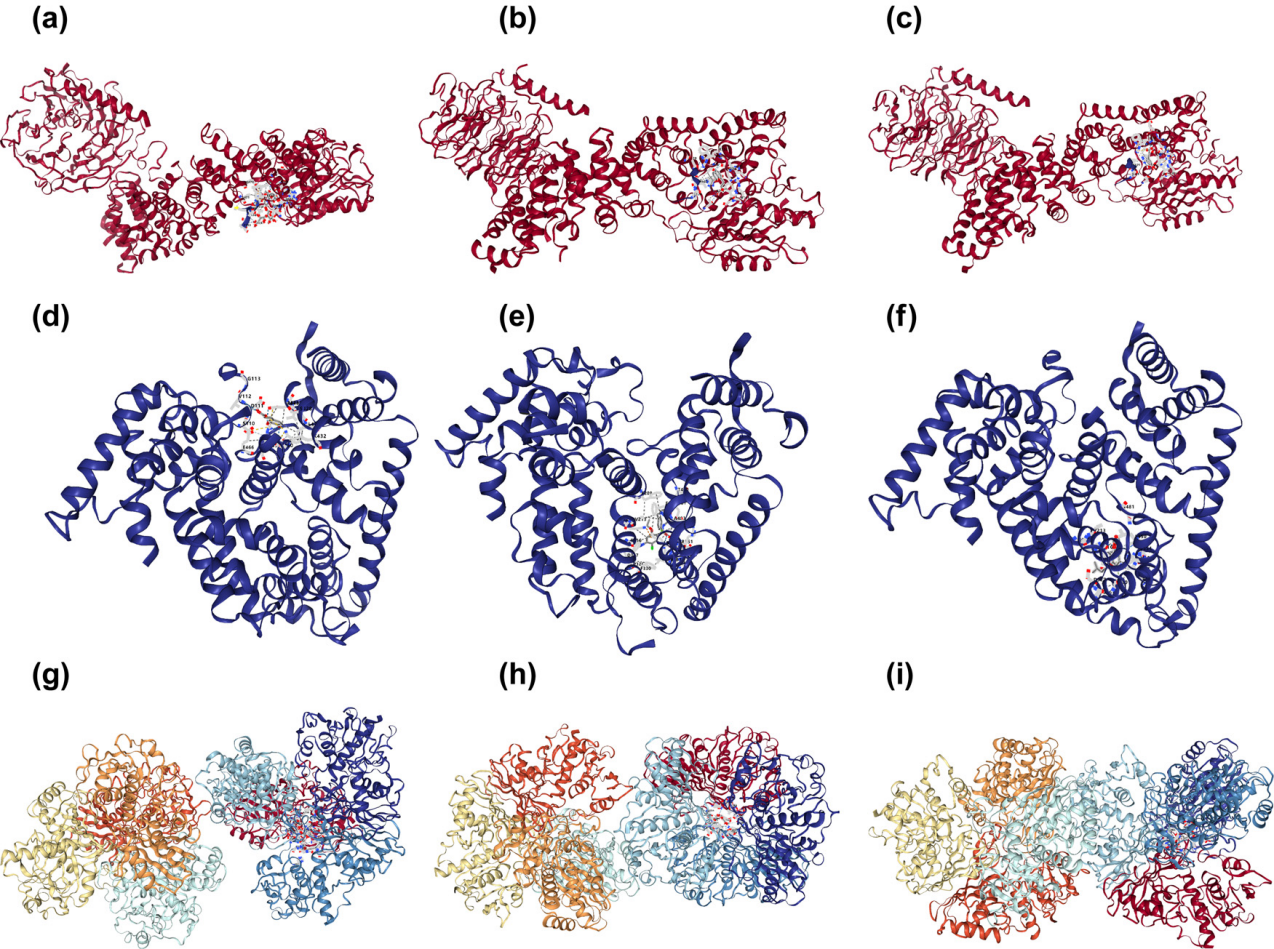
Molecular docking results: (a) AKT1 was docked to quercetin, (b) AKT1 was docked to kaempferol, (c) AKT1 was docked tobaicalein, (d) JUN was docked to quercetin, (e) JUN was docked to beta-sitosterol, (f) JUN was docked to kaempferol, (g) RELA was docked to quercetin, (h) RELA was docked to kaempferol, and (i) RELA was docked to wogonin.

It is commonly accepted that the more stable the conformation of ligand–receptor interaction and the greater the likelihood of action, the lower the energy. The core proteins (JUN, AKT1, and RELA) screened in “2.5” were molecularly docked with the core components screened in “2.4.” It is generally believed that the drug molecules with binding energy less than −5.00 kJ/mol have better binding activity with the proteins (Figure 7). As shown in Table 2, JUN, AKT1, and RELA had better binding activity with quercetin and kaempferol. The above results indirectly proved that the active main components of HLJDD can exert regulatory effects on core proteins.

**Table 2:** Binding energy values of the key compounds in HLJDD and targets

Protein	Binding energy (kJ/mol)				
	MOL000098 (quercetin)	MOL000358 (beta-sitosterol)	MOL000422 (kaempferol)	MOL002714 (baicalein)	MOL000173 (wogonin)
JUN	−11.8	−9.5	−7.0	—	—
AKT1	−10.8	—	−10.5	−9.7	—
RELA	−9.4	—	−8.7	—	−10.1

## 4 Discussion

COVID-19 pneumonia is still in the outbreak phase worldwide. In addition to causing respiratory symptoms, COVID-19-associated AKI is also a widespread concern. One study from the Journal of the American Society of Nephrology (2021) found that patients with COVID-19 pneumonia who were not hospitalized for treatment had a 23% increased risk of AKI within 6 months after comparing the physical status of 89,216 people who recovered from COVID-19 pneumonia to 1,637,467 people who were not infected [30]. Patients with COVID-19 are at significant risk for renal impairment, and the need for attention in the post-acute care of COVID-19 is emphasized. Therefore, it is particularly important how to prevent and treat COVID-19-associated AKI.

The National Health Commission and other health organizations in numerous locations have sequentially released a variety of diagnoses and treatment regimens since the COVID-19 pneumonia outbreak [10,31]. The “epidemic Qi” of TCM and COVID-19 both have comparable pathogenicity [32]. “Heat-toxin obstruction in the lungs” and “Qi dysfunction in the fu-organs” are the primary pathophysiology. In classical Chinese philosophy, “fire” is one of the five “basic elements” (wood, fire, earth, gold, and water), a seemingly contradictory dual elemental role [33]. Excessive amount of “body fire” can have harmful effects and form the basis for many diseases. According to the “four nature theory,” all Chinese herbs are divided into four categories, including “cold,” “hot,” “warm,” and “cool” herbs. Based on this theory, all four herbs in HLJDD are considered “heat-clearing” herbs, which means that they can completely remove the “body fire” and erase the pattern of excess heat-toxicity in “triple-jiao” [32–35]. In fact, the essence of “body fire” is a gradual process including oxidative/nitrosative stress, inflammation, and infection. Inflammatory processes can damage cells in multiple parts of the body. Cytokine storms may be a key sign of the deterioration of COVID-19 [36]. The key to the cytokine storm is the link between inflammation and oxidative stress. These two processes reinforce each other and trigger a vicious cycle.

The exact mechanism of COVID-19-associated AKI has not been fully understood [4–6]. There is evidence of direct involvement of the SARS-CoV-2 virus in the renal tubular epithelium. The virus binds to ACE2. ACE2 is expressed more in gastrointestinal organs and kidneys than in the lungs, implying that the kidneys are susceptible to viral infection and injury [37]. One study analyzed kidney abnormalities in 26 autopsies of patients with COVID-19 by light microscopy, ultrastructural observation, and immunostaining. Electron microscopic examination showed clusters of coronavirus-like particles with distinctive spikes in the tubular epithelium and podocytes [38]. These results provided direct evidence for the invasion of SARS-CoV-2b into renal tissue, but the direct role of this virus in the development of AKI remain to be confirmed. Immune activation-mediated invasive inflammation (even cytokine storms) and inadequate blood oxygen supply contributed to the development of acute lung injury and ARDS [39]. The elevation of IL-7, granulocyte-macrophage colony stimulating factor, interferon-gamma (IFN-γ), and fibroblast growth factor in COVID-19 patients might be involved in the development of AKI in COVID-19 patients and induced endothelial cell and tubular dysfunction [40]. Other possible mechanisms included “Organ Crosstalk” [41], “complement activation” [42], and “downregulation of ACE2 expression” [43].

According to “Novel Coronavirus Pneumonia Diagnosis and Treatment Plan (Trial Operation Seventh Edition),” HLJDD is one of the prescriptions recommended for COVID-19 pneumonia that is in its most severe phases [31]. The study of HLJDD has good clinical value for the treatment of severe COVID-19 and related complications. In this study, we investigated the mechanism of HLJDD in the treatment of COVID-19-associated AKI based on network pharmacology and molecular docking. The active compounds–targets network diagram was constructed, and the results showed that quercetin and kaempferol were the main chemical components of the herbal compound. The proportion of quercetin was 71/353, 20.11%, while the proportion of kaempferol was 24/353, 6.8%. It was found that quercetin and kaempferol are the key compounds in the “Three Chinese Medicines and Three Chinese Recipes” for COVID-19 treatment [9]. Modern research studies show that quercetin is widely distributed in plants as a flavonol compound with a variety of biological effects such as antioxidant [35], anti-inflammatory [44], anti-tumor [45], antiviral [46], and immunomodulatory [44]. Quercetin inhibits mast cell-mediated inflammatory responses and exerts anti-inflammatory effects by acting on NF-κB and TLR4 signaling pathways and inhibiting the release of related inflammatory mediators [47]. A network pharmacology study found a protective effect of quercetin in COVID-19-induced AKI, revealing a possible pathological mechanism of kidney injury during coronavirus disease [20]. Kaempferol is a flavonol compound with an antibacterial, anti-inflammatory, cough suppressant, expectorant, and immune function enhancing effects [48]. Kaempferol has a significant inhibitory effect on the inflammatory response of mast cells [49]. Kaempferol reduces oxidative stress and inflammation in the kidney via the AR/NOX2 pathway, thereby promoting renal cell growth activity and improving renal injury [50]. At the onset of COVID-19, the integrity of the air–blood barrier between the blood vessels and alveoli of the lungs is reduced and a large number of inflammatory cytokines are released thereby triggering an inflammatory response. The kidney undergoes an inflammatory response as well as oxidative stress in patients with COVID-19 combined with renal injury. HLJDD inhibits inflammatory reactions and reduces oxidative stress through quercetin, kaempferol, and other effective chemical components to achieve therapeutic purposes.

Our study suggests that HLJDD can treat disease through the effective active components acting on the genes of JUN, RELA, and AKT1 together. C-Jun can activate NLRP3 inflammatory vesicles, promote phosphorylation, and mediate inflammatory responses [51]. RELA, also known as NFKB3, encodes the 551 amino acid NFKB transcription factor [52]. It can specifically bind to promoter and enhancer lust sites of many genes, regulate gene transcription and expression, participate in inflammation [53], immune [54], oxidative stress [55], and other responses. It can regulate cell differentiation, proliferation, apoptosis, and other processes [56]. AKT1 is one of the serine/threonine protein kinases, and AKT is a central factor in the PI3K/AKT signaling pathway involved in regulating cell growth and metabolism, proliferation and migration, and angiogenesis [57]. In clinical practice, patients with COVID-19 and AKI ultimately have life-threatening effects from pulmonary fibrosis and renal fibrosis. Abnormal activation of PI3K/AKT/mTOR pathway is important in fibrotic diseases because mTOR signaling not only regulates cellular autophagy but also pulmonary fibrosis, and inhibition of PI3K/AKT/mTOR signaling pathway can have an anti-pulmonary fibrosis effect [58].

The GO analysis suggested that cytokine and receptor binding and receptor ligand activity were the main biological processes. The KEGG pathway enrichment analysis showed that these overlapping genes were mainly enriched in pathways, such as lipid and atherosclerosis signaling pathway, IL-17 signaling pathway, and AGE-RACE signaling pathway. Previous studies have shown that atherosclerosis can cause luminal narrowing and a chronic inflammatory response in the vascular wall. The pathogenesis of COVID-19-associated AKI was based on damage to the pulmonary and renal capillaries, and abnormalities in lipid and atherosclerotic signaling pathways lead to acute inflammation of the pulmonary and renal capillaries and thus become the basis for pathogenesis. IL-17 can activate the NF-κB and MAPK pathways. Both NF-κB and MAPK are target genes for lL-17 to exert their pro-inflammatory effects, and the IL-17 signaling pathway plays a crucial role in host defense against microbes and the development of inflammatory diseases [59,60]. AGE-RAGE activates nuclear transcription factor (NF-κB), which has been shown to lead to the expression and release of a large number of adhesion molecules, growth factors, and pro-inflammatory cytokines [61]. In the pathogenesis of COVID-19-associated AKI, an inflammatory response occurs and activates the AGE-RAGE signaling pathway. Blocking the AGE-RAGE signaling pathway can effectively inhibit the release of inflammatory factors and achieve the purpose of treating the disease [62].

Quercetin and kaempferol were the main components in HLJDD, while JUN, RELA, and AKT1 were the core proteins in the PPI network. The molecular docking validation showed that the lowest binding energies of molecules to proteins were all less than −5.00 kJ/mol, indicating that both active ingredients had good binding activities with JUN, RELA, and AKT1, which was the same as the results of the previous study [63–65].

## 5 Conclusion

This study combined the network pharmacology and bioinformatics to systematically analyze and predict the compound–target pathway mechanism of the action of HLJDD for the treatment of COVID-19-associated AKI. The potential mechanisms of HLJDD in treating COVID-19-associated AKI may be related to anti-inflammation, lipid lowering, counteracting oxidative stress, promoting apoptosis of senescent cells, and repairing damage. These findings may provide potential references and theoretical aspects for the clinical application of HLJDD. However, the network pharmacology has limitations and the pathogenic mechanism of COVID-19-associated AKI is not fully understood at present. Further validations through experiments and clinical practice are needed. The challenge ahead is to translate these potential preclinical discoveries and develop effective drugs for the treatment of COVID-19 and its complications.

## Abbreviations

ACE2: angiotensin-converting enzyme II
AKI: acute kidney injury
ARF: acute renal failure
COVID-19: Corona virus disease 2019
GO: gene ontology
HLJDD: Huanglian Jiedu Decoction
KEGG: Kyoto Encyclopedia of genes and genomes
PPI: protein–protein interaction
TCM: traditional Chinese medicine
